# Exploring the molecular mechanism underlying the psoriasis and T2D by using microarray data analysis

**DOI:** 10.1038/s41598-023-46795-5

**Published:** 2023-11-07

**Authors:** Li Yang, Lei Zhang, Qingfang Du, Xiaoyu Gong, Jun Tian

**Affiliations:** 1 Department of Dermatology, Shaanxi Provincial People’s Hospital, Xi’an, China; 2 Department of Ophthalmology, Shaanxi Provincial People’s Hospital, Xi’an, China

**Keywords:** Cancer, Molecular biology

## Abstract

Although a large number of evidence has identified that psoriasis is significantly correlated with type 2 diabetes (T2D), the common molecular mechanism of its occurrence remains unclear. Our study aims to further elucidate the mechanism of the occurrence of this complication. We obtained the gene expression data of psoriasis (GSE30999) and T2D (GSE28829) from the Gene Expression Omnibus (GEO) dataset. Then the common differentially expressed genes (DEGs) of T2D and psoriasis were identified. After that, we performed three types of analyses about these DEGs, including functional enrichment analysis, protein–protein interaction (PPI) network and module manufacture, hub genes identification and co-expression analysis. 132 common DEGs (14 upregulated genes and 118 downregulated genes) were identified for subsequent a series of analyses. Function enrichment analysis demonstrated that Rap1 signaling pathway, PI3K-Akt signaling pathway, and cGMP-PKG signaling pathway may play a significant role in pathogenesis of psoriasis and T2D. Finally, 3 important hub genes were selected by utilizing cytoHubba, including SNRPN, GNAS, IGF2. Our work reveals the potential common signaling pathways of psoriasis and T2D. These Hub genes and common signaling pathways provide insights for further investigation of molecular mechanism about psoriasis and T2D.

## Introduction

Psoriasis and type 2 diabetes (T2D) are prevalent chronic diseases with a significant global impact. Psoriasis is characterized by chronic inflammation and abnormal keratinocyte proliferation in the skin, and the prevalence of psoriasis varies across different population and geographical regions. In general, psoriasis affects around 2–3% of the population in Western countries, with higher rates reported in some population, such as those in Scandinavia and the Caucasus region^1^. The condition can occur at any age, but onset is most common in the second to fourth decades of life. T2D, a metabolic disorder that compromises the body's ability to regulate blood glucose levels, is also a growing global health concern, with an estimated 463 million patients^2^. Although these diseases affect different organ systems, recent epidemiological evidence highlights a significant association between psoriasis and T2D, indicating shared molecular mechanism^3^.

Multiple studies suggest that psoriasis may increase the risk of developing diabetes^3^. Individuals with psoriasis have a higher prevalence of diabetes than those without, and those with severe psoriasis have an increased incidence of diabetes. Moreover, psoriasis may increase susceptibility to insulin resistance, a prelude to type 2 diabetes^4^. Conversely, diabetes may increase the risk of developing psoriasis, particularly for those with poorly controlled blood glucose levels. The relationship between psoriasis and diabetes appears to be complex and possibly bidirectional, with chronic inflammation and immune dysregulation playing a critical role in the pathogenesis of both diseases.

One common pathway in both conditions is the activation of the nuclear factor kappa-light-chain-enhancer of activated B cells (NF-κB) signaling pathway. In psoriasis, NF-κB activation culminates in the upregulation of proinflammatory cytokines, chemokines, and adhesion molecules that promote immune cell infiltration and inflammation^5^. Similarly, in diabetes, NF-κB activation contributes to chronic low-grade inflammation and insulin resistance^6^. Chronic inflammation in psoriasis leads to the release of pro-inflammatory cytokines that can interfere with insulin signaling, leading to insulin resistance and impaired glucose metabolism, which are hallmarks of the T2D^7^. Insulin resistance can further contribute to the progression of psoriasis by promoting inflammation, oxidative stress, and aberrant lipid metabolism^8^. However, the association between psoriasis and T2D is complex and multifactorial. Besides chronic inflammation, insulin resistance and shared risk factors such as obesity and smoking are also believed to be the mechanisms underlying this association^9,10^. A multidisciplinary approach is essential to manage these chronic conditions effectively, addressing shared risk factors and improving overall metabolic health.

The purpose of this study is to elucidate the molecular underpinnings of psoriasis and T2D using a systems biology approach based on microarray data analysis. We analyzed gene expression data from skin biopsies and blood samples of patients with psoriasis, T2D, or both, as well as healthy controls. We used various bioinformatics tools to highlight differentially expressed genes (DEGs), enrich gene ontology, and conducted a network analysis to identify critical regulatory pathways. Through this study, we could better understand the molecular mechanisms underlying the association between psoriasis and T2D. Ultimately, our study may provide novel therapeutic targets, potentially revolutionizing the way we approach these diseases and opening new horizons in medical treatment.

## Material and methods

### Data collection

GEO is an invaluable public repository that houses an extensive collection of high-throughput sequencing and microarray data sets, contributed by worldwide research institutions^11^. In order to identify gene expression datasets relevant to our study, we conducted a search using the keywords “psoriasis” and “T2D”. Our inclusion criteria were defined as follows: selecting two separate expression profiles, both originating from the same sequencing platform; prioritizing the datasets with the largest sample size, and ensuring that all data was derived exclusively from human specimen. By adhering to these parameters, we successfully obtained two microarray datasets, namely GSE30999 and GSE25724, from the GEO database. Both datasets were obtained using the Affymetrix GPL570 platform. The GSE30999 dataset comprises 85 paired samples from patients with psoriasis, consisting of skin lesions (LS) and adjacent normal tissues (NL). These samples provide a valuable resource for investigating the gene expression patterns associated with psoriasis. On the other hand, the GSE25724 dataset includes 6 pancreatic islet tissues of T2D and 7 non-diabetic isolated human islet samples.

### Identification of DEGs

GEO2R is a powerful online tool designed for gene expression analysis. This tool utilizes GEOquery and Limma R packages^12^. The GEOquery package enables data retrieval from the GEO database, while the Limma package is employed for the calculation of differential expression multiples. The workflow of GEO2R involves comparing gene expression profiles between distinct groups, typically a diseased group and a control group, in order to identify the DEGs. Prior to analysis, probe sets lacking corresponding gene symbols are eliminated. Genes associated with multiple probe sets are either averaged or processed in a suitable manner. To determine the DEGs, GEO2R applies specific criteria. Only genes meeting two conditions are considered: a P-value below 0.05, suggesting statistical significance, and an absolute fold change (|logFC|) greater than or equal to 1, indicating a substantial difference in gene expression. Genes satisfying these criteria are identified as DEGs. GEO2R also employs an online Venn diagram tool to generate a graphical representation of the common DEGs shared between different groups, allowing for a comprehensive comparison and analysis.

### Function enrichment analyses of DEGs

Gene ontology (GO) is a comprehensive resource that was developed by the Gene ontology federation. Its primary purpose is to provide straightforward annotations of gene products, including their functions, involvement in biological pathways, and cellular locations. GO serves as a valuable resource for researchers studying the molecular aspects of genes. Complementing GO is another valuable database known as the Kyoto Encyclopedia of Genes and Genomes (KEGG) pathway^13–15^. This resource focuses on the compilation of gene pathways from a range of species, thereby enhancing our understanding of intricate biological processes and the regulatory mechanisms governing them. By storing information about gene pathways, KEGG pathway analysis facilitates the understanding of complex biological processes and their regulatory mechanisms. Our research leveraged both the KEGG and GO databases, utilizing the “clusterProfiler” package in R for the analysis. The resultant data were then visualized with the aid of the “ggplot2” package, enhancing the clarity and understanding of our findings.

### PPI network construction and module analysis

The search tool for the retrieval of interacting genes (STRING) is a tool that allows for in-depth exploration of the relationships among proteins^16^. It enables users to investigate various types of interactions, including direct binding relationships and concurrent upstream and downstream regulatory mechanisms. By utilizing STRING, we can facilitate the creation of a Protein–Protein Interaction (PPI) network that captures the complex regulatory interplay between proteins. The interactions possessing a combined score greater than 0.4 are deemed statistically significant. To visually represent the constructed PPI network, Cytoscape was employed along with its plugin, the molecular complex detection technology (MCODE). MCODE enables the analysis of principal functional modules within PPI network^17^. To identify the significant functional modules, specific selection criteria are set within MCODE. These criteria include a K-core value of 2, a degree cutoff of 2, a maximum depth of 100, and a node score cutoff of 0.2.

### Selection and analysis of Hub genes

The identification of hub genes was performed by utilizing the cytoHubba plug-in within the Cytoscape software. In this analysis, we employed seven widely recognized algorithms, namely MCC, Degree, MNC, Radiality, Closeness, EPC and Stress, for a comprehensive evaluation and precise selection of the most significant hub genes. To further explore internal associations within selected hub genes, we designed a co-expression network utilizing GeneMANIA platform. GeneMANIA, acclaimed for its reliability, enables the identification of reliable associations among gene sets^18^.

### Validation of hub genes expression

The mRNA expression levels of the pinpointed hub genes were confirmed using GSE14905. In the GSE14905 dataset, there were 33 samples of LS, 28 samples of NL, and 21 samples of normal skin (NS). To compare the gene expression patterns of this dataset, a *T*-test was conducted. A P-value below 0.05 was identified for statistical significance.

### Clinical sample collection

5 peripheral blood samples of T2D patients and 5 healthy control samples were collected for qRT-PCR experiment to validate hub genes expression. These samples were obtained from Shaanxi Provincial People’s Hospital. All experiments were performed as per the guidelines recommended by the Ethics Approval Committee of the institute. Red blood cell lysis solution was utilized to lyse red blood cells. Then we collected cell precipitates by centrifugation at 500 rpm for 10 min, which were further lysed with TRIzol Reagent. RNA was extracted and purified using chloroform, isopropanol, and ethanol solutions. Reverse transcription kit and SYBR qPCR Master Mix (Shandong Sparkjade Biotechnology Co., Ltd.) were utilized for the cDNA synthesis of the target genes according to the manufacturer’s instructions. The qRT − PCR primers were as follows: SNRPN, forward 5′-CGGGCAAGGGATCGCTTAC-3′ and reverse 5′-GGGTACAACTGACACTCTTGG-3′; GNAS, forward 5′-CGGAGCCCCAGATAAGAGAGA-3′ and reverse 5′-CCCGGAGAGGGTACTTTTCCT-3′. IGF2, forward 5′-GTGGCATCGTTGAGGAGTG-3′ and reverse 5′-CACGTCCCTCTCGGACTTG-3′.

### Prediction and verification of transcription factors (TFs)

Transcriptional regulatory relationships unraveled by sentencebased text mining (TRRUST) database is a powerful tool designed to predict transcriptional regulatory networks^19^. It encompasses target genes associated with TFs as well as the existing regulatory relationships between them primarily focusing on human and mouse species. Within the database, there are a comprehensive collection of regulatory relationships involving 800 TFs and their target genes in the human species, and 828 TFs and their target genes in the mouse species. To identify TFs that regulate hub genes, we employed the TRRUST database. We considered a regulatory relationship significant if it met the criterion of an adjusted P-value less than 0.05. Following this, we conducted further analysis by examining the expression levels of these identified TFs across two datasets: GSE30999 and GSE25724. Significance of the differences in expression levels was assessed using the *T*-test.

## Results

### Identification of DEGs

**Figure 1:**
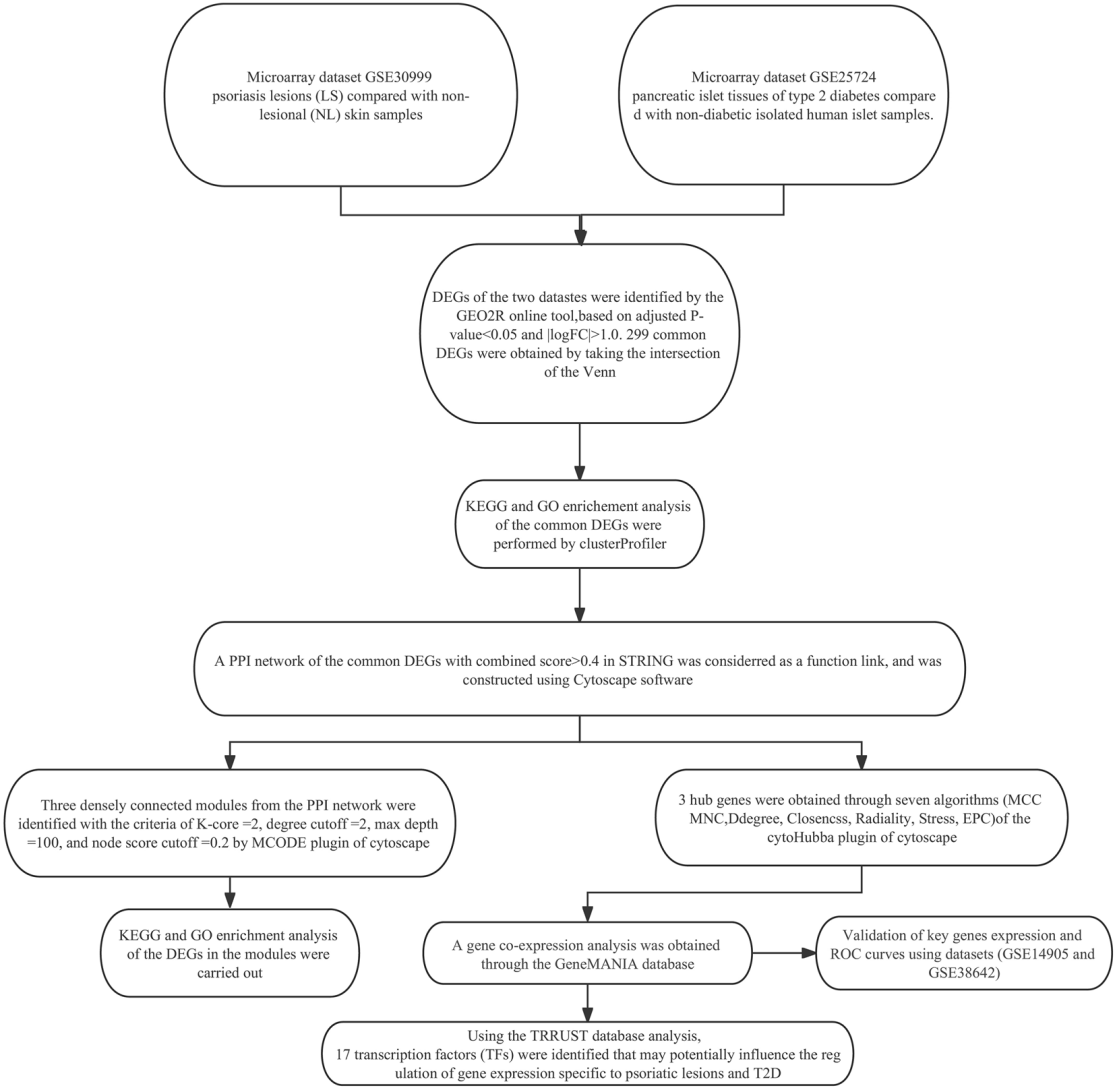
Research design flow chart.

**Figure 2:**
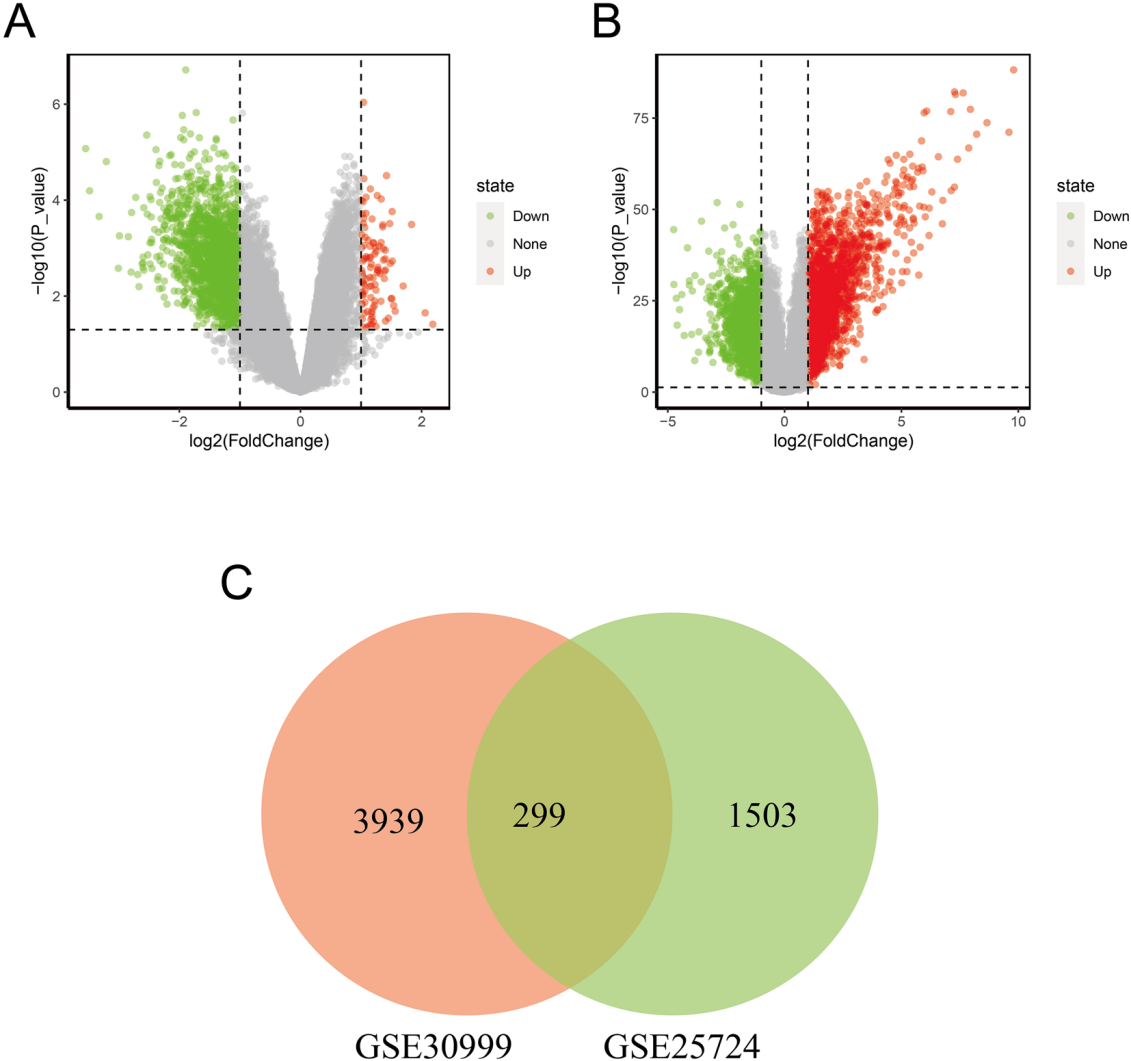
Volcano diagram and Venn diagram. (**A**) The volcano plot of GSE30999. (**B**) The volcano plot of GSE25724. The light red and light green points represent up-regulated and down-regulated genes, respectively. (**C**) Overlap of 299 DEGs from both datasets.

The research flowchart of our study was displayed in Fig. 1. Following the standardization of the microarray data, we identified DEGs in two independent datasets: GSE30999 (4238 DEGs) and GSE25724 (1802 DEGs) (Fig. 2A,B). To unearth the common DEGs present within both datasets, we performed a Venn diagram analysis and obtained 299 genes significantly differentially expressed in both datasets (Fig. 2C). These results suggested a robust set of genes consistently differentially expressed across multiple datasets and may have important functional implications. Subsequently, 132 DEGs were obtained after excluding genes which had adverse expression trends in GSE25724 and GSE30999, containing 14 upregulated genes and 118 downregulated genes.

### Analysis of the functional characteristics of DEGs

**Figure 3:**
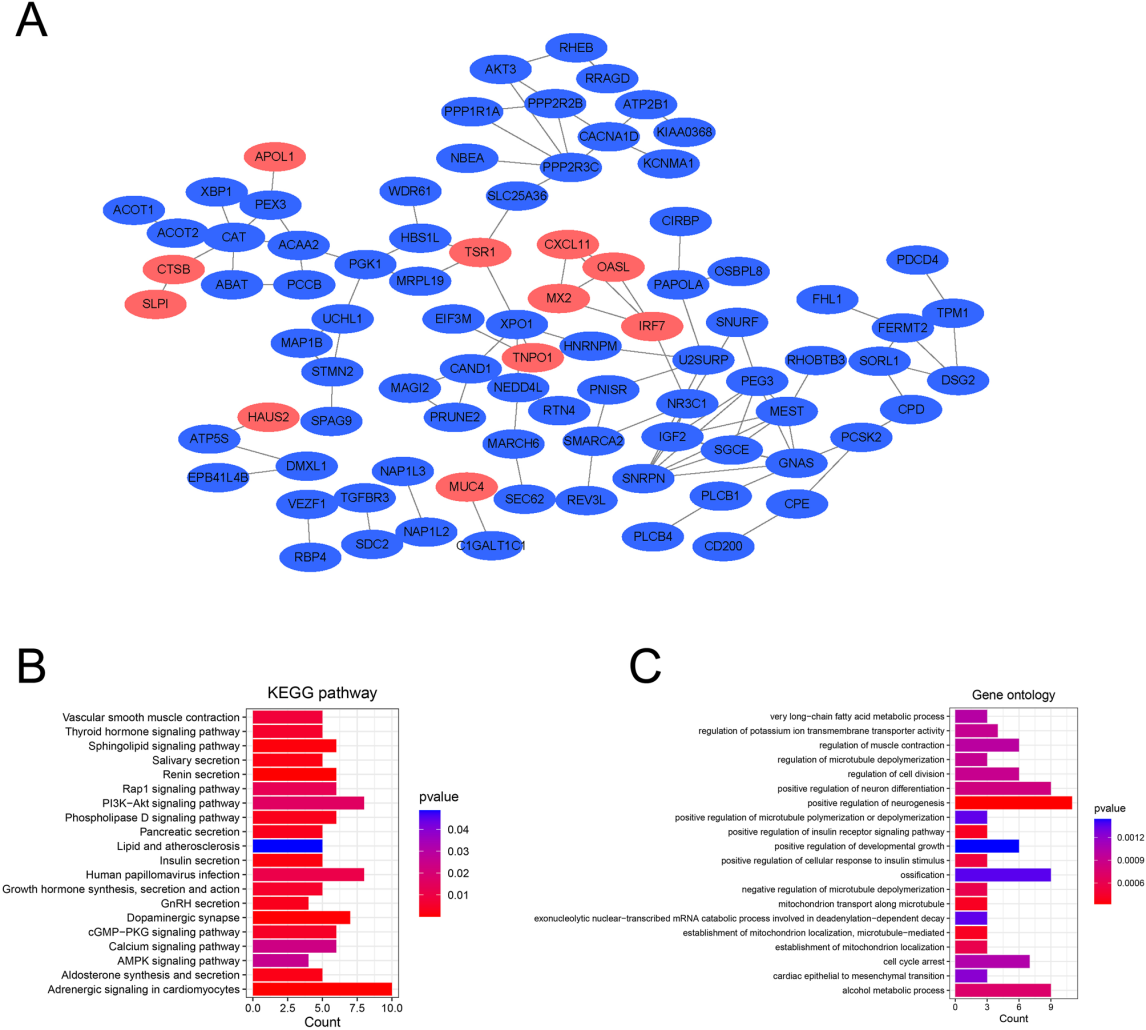
Results of the PPI network and enrichment analysis of shared DEGs. (**A**) Visualization of the PPI network. Red nodes represent up-regulated genes and blue-violet nodes are down-regulated genes. (**B**,**C**) Outcomes of GO and KEGG pathway enrichment analysis. An adjusted P-value of less than 0.05 was considered statistically significant.

The PPI network was constructed utilizing Cytoscape based on DEGs that exhibited combined scores exceeding 0.4, resulting in 87 nodes and 107 interaction pairs (Fig. 3A). In the exploration of the biological functions and pathways involved in the 299 shared differentially expressed genes (DEGs), we conducted GO and KEGG pathway enrichment analyses. The KEGG pathway analysis revealed several significant enrichment pathways, including vascular smooth muscle contraction, thyroid hormone signaling pathway, sphingolipid signaling pathway, salivary secretion, renin secretion, Rap1 signaling pathway, phospholipase D signaling pathway, PI3K-Akt signaling pathway, pancreatic secretion, growth hormone synthesis, secretion, and action, dopaminergic synapse, cGMP-PKG signaling pathway, calcium signaling pathway, aldosterone synthesis and secretion and adrenergic signaling in cardiomyocytes (Fig. 3B). The GO analysis results revealed significant enrichment in several biological processes, including regulation of muscle contraction, regulation of cell division, positive regulation of neuron differentiation, positive regulation of neurogenesis, positive regulation of developmental growth, ossification, cell cycle arrest, and alcohol metabolic process (Fig. 3C). These findings strongly suggested that the pathogenesis of psoriasis and type 2 diabetes involves multiple physiological processes, including cellular signaling, metabolic processes, and neurological functions.

### PPI network construction and functional enrichment analysis

**Figure 4:**
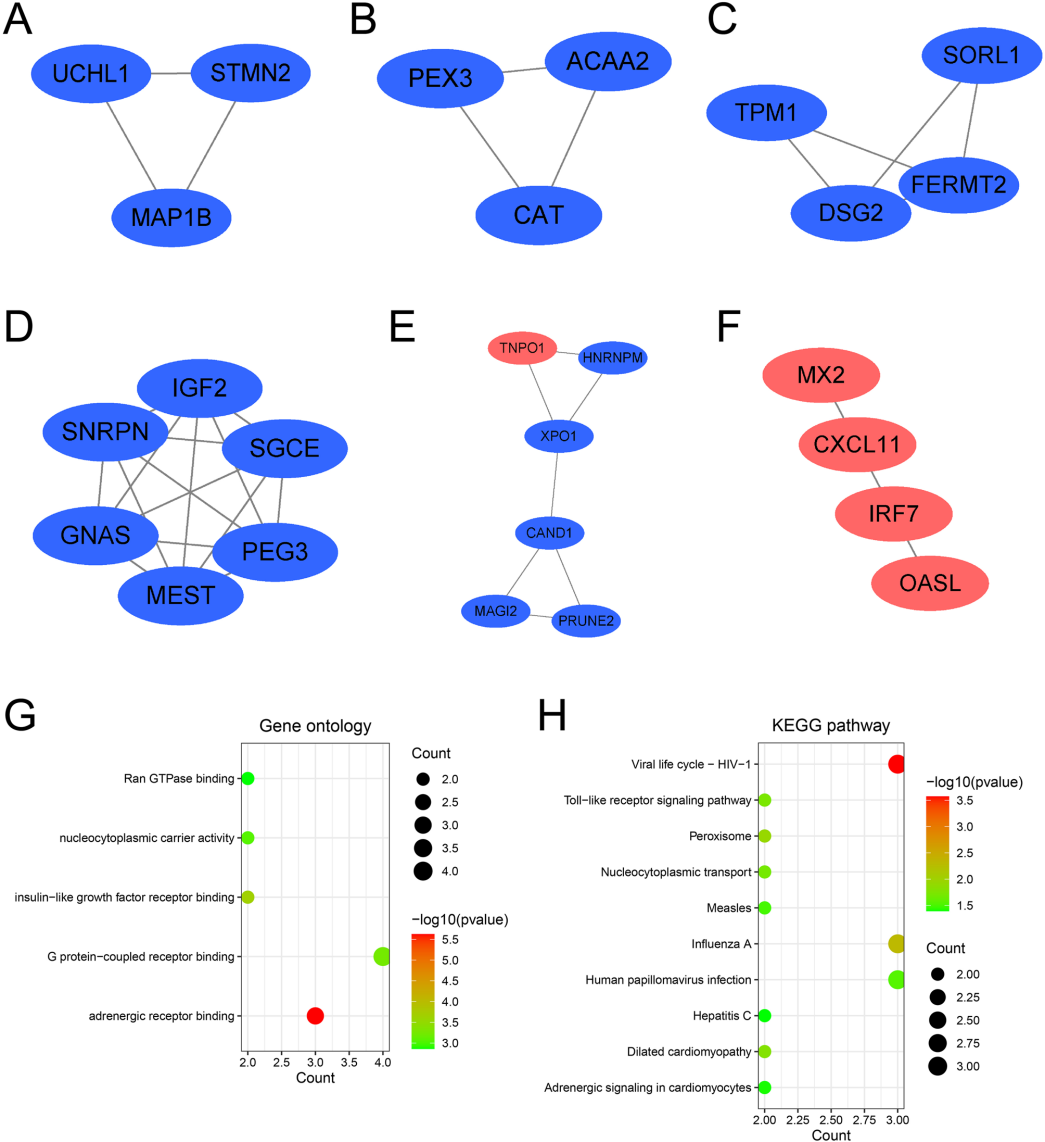
Significant gene module and enrichment analysis of the modular genes. (**A**–**F**) Identification of six key gene clustering modules. (**G**,**H**) Exploration of GO and KEGG enrichment. The size of each circle corresponds to the number of genes involved within each term or pathway. The abscissa represents the relative frequency of genes within each term or pathway with respect to the total number of genes under study.

Six highly interconnected gene modules were identified using the MCODE plug-in within Cytoscape, comprising 26 shared DEGs and 39 interaction pairs (Fig. 4A–F). GO analysis revealed that the identified genes mainly participated in G protein-coupled receptor binding and adrenergic receptor binding (Fig. 4G). KEGG pathway analysis, on the other hand, pointed out that these genes predominantly played a role in viral life cycle-HIV-1, influenza A, and human papillomavirus (Fig. 4H), suggesting that these genes were closely correlated with virus infection.

### Screen and analysis of hub genes

**Figure 5:**
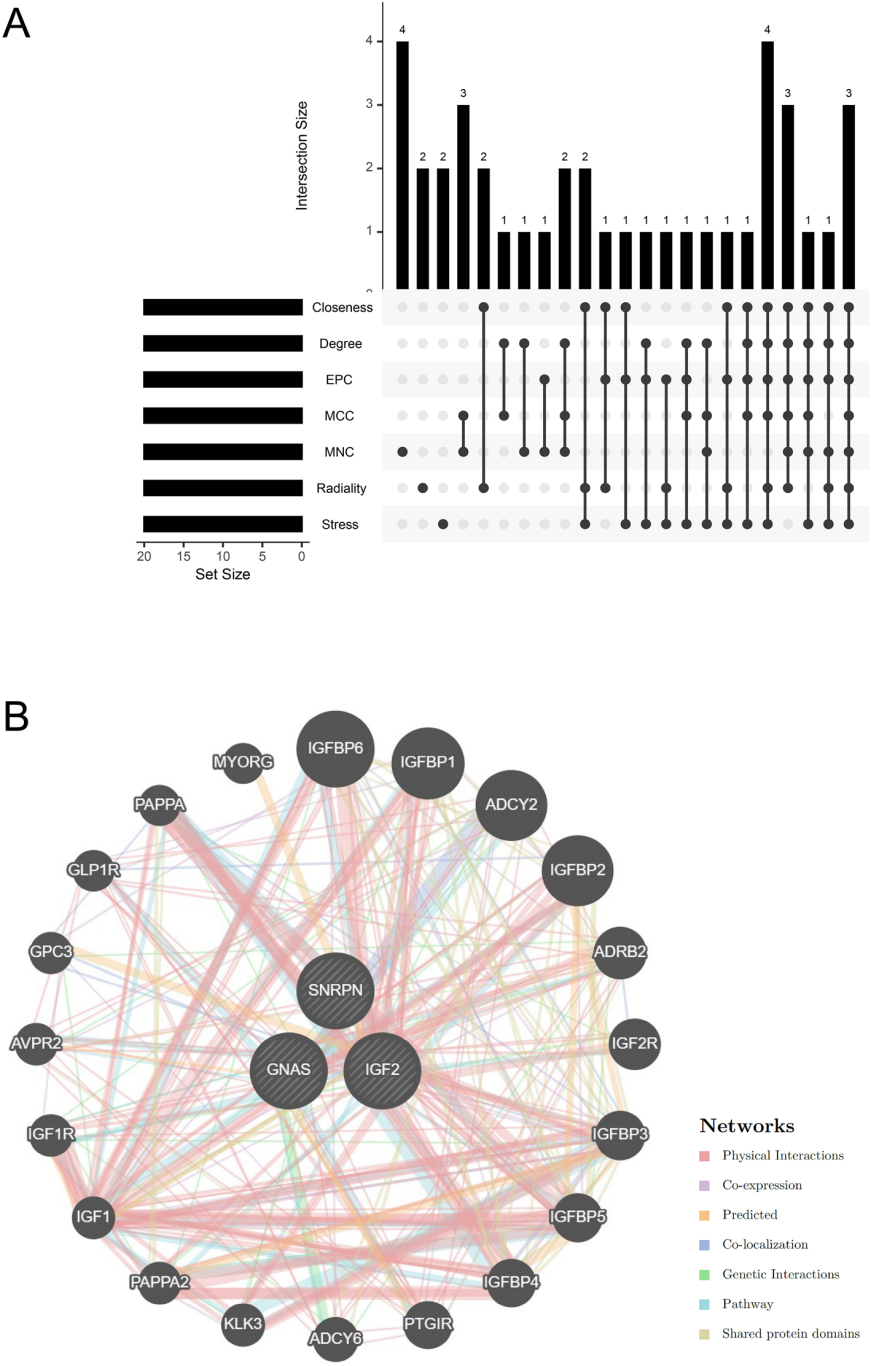
Upset plot and co-expression network of hub genes. (**A**) The upset plot illustrated the intersection of seven different algorithms, reveals the identification of three common hub genes. (**B**) Hub genes and their corresponding co-expression genes were analyzed by utilizing GeneMANIA tool.

**Figure 6:**
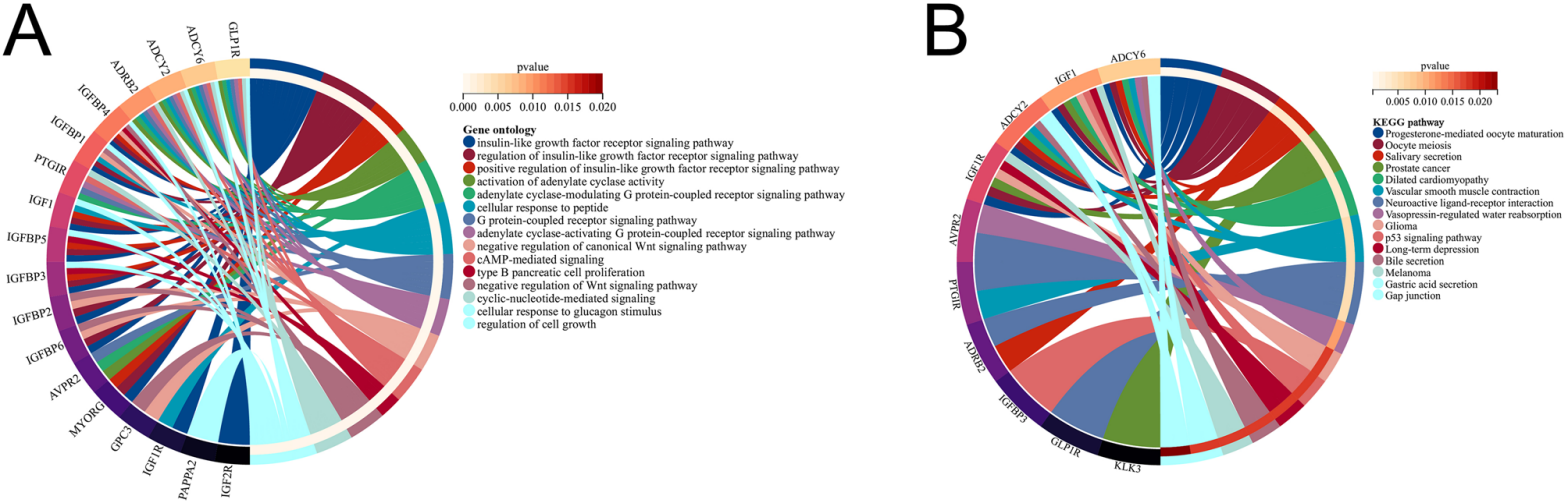
Enrichment analysis of the hub genes (**A**,**B**) GO and KEGG enrichment analysis of the hub genes. The peripheral circle on the right and the inner circle on the left represent the statistical significance of the corresponding P-value of the pathway associated with the gene.

We utilized the cytoHubba plug-in and applied seven algorithms to identify the top 20 hub genes in our study (Table 1). By examining the overlap within Venn diagrams, we identified 3 shared hub genes, including SNRPN, GNAS, and IGF2 (Fig. 5A). To gain further insight into the potential functions of these genes, we investigated their co-expression network and related functions utilizing the GeneMANIA database. Our analysis revealed a sophisticated PPI network, characterized by 77.64% physical interactions, 8.01% co-expression levels, 5.37% predicted interactions, 3.63% co-localization, 2.87% genetic interactions, and 1.88% pathway interactions (Fig. 5B). The expression of SNRPN, GNAS, and IGF2 was closely associated with 20 genes, including IGFBP6, IGFBP1, ADCY2, IGFBP2, and so on. In addition, we carried out GO and KEGG analysis about these 20 genes associated with SNRPN, GNAS, and IGF2. GO analysis revealed their involvement in diverse biological processes, including the insulin-like growth factor receptor signaling pathway, adenylate cyclase activation, G protein-coupled receptor signaling pathway, negative regulation of canonical Wnt signaling pathway, and cAMP-mediated signaling (Fig. 6A). These results suggested the significant role of the G protein-coupled receptor signaling pathway, Wnt signaling pathway, and cAMP-mediated signaling in these two diseases. KEGG pathway analysis demonstrated that these genes were prominently enriched in progesterone-mediated oocyte maturation, oocyte meiosis, salivary secretion, prostate cancer, and dilated cardiomyopathy (Fig. 6B).

**Table 1:** The top 20 hub genes identified by seven algorithms.

MCC	MNC	Degree	Closeness	Radiality	Stress	EPC
SNRPN	PEG3	GNAS	SNRPN	XPO1	TSR1	GNAS
GNAS	SNRPN	SNRPN	XPO1	HNRNPM	XPO1	SNRPN
PEG3	GNAS	CAT	U2SURP	TSR1	U2SURP	IGF2
MEST	MEST	PPP2R3C	GNAS	U2SURP	HNRNPM	PEG3
IGF2	SGCE	MEST	TSR1	TNPO1	PGK1	MEST
SGCE	IGF2	PEG3	IGF2	SNRPN	SNRPN	SGCE
PPP2R3C	PPP2R3C	IGF2	HNRNPM	HBS1L	ACAA2	U2SURP
IRF7	PPP2R2B	XPO1	MEST	MRPL19	GNAS	SNURF
CAT	CXCL11	SGCE	PEG3	SLC25A36	HBS1L	XPO1
PPP2R2B	OASL	TSR1	SGCE	NEDD4L	MRPL19	HNRNPM
CXCL11	MX2	U2SURP	HBS1L	CAND1	SLC25A36	NR3C1
OASL	IRF7	ACAA2	PGK1	PNISR	PCSK2	TSR1
MX2	DSG2	PGK1	TNPO1	GNAS	PPP2R3C	PCSK2
XPO1	FERMT2	PPP2R2B	PPP2R3C	IGF2	CAT	PGK1
FERMT2	HNRNPM	CACNA1D	SLC25A36	PAPOLA	CPD	TNPO1
TSR1	MAP1B	IRF7	MRPL19	PGK1	NR3C1	ACAA2
U2SURP	UCHL1	FERMT2	NEDD4L	MEST	SORL1	HBS1L
ACAA2	STMN2	HNRNPM	ACAA2	PEG3	IGF2	PPP2R3C
PGK1	SNURF	NR3C1	CAND1	SGCE	IRF7	CAT
CACNA1D	PPP1R1A	UCHL1	PCSK2	EIF3M	PNISR	PNISR

### Validation of hub genes expression

**Figure 7:**
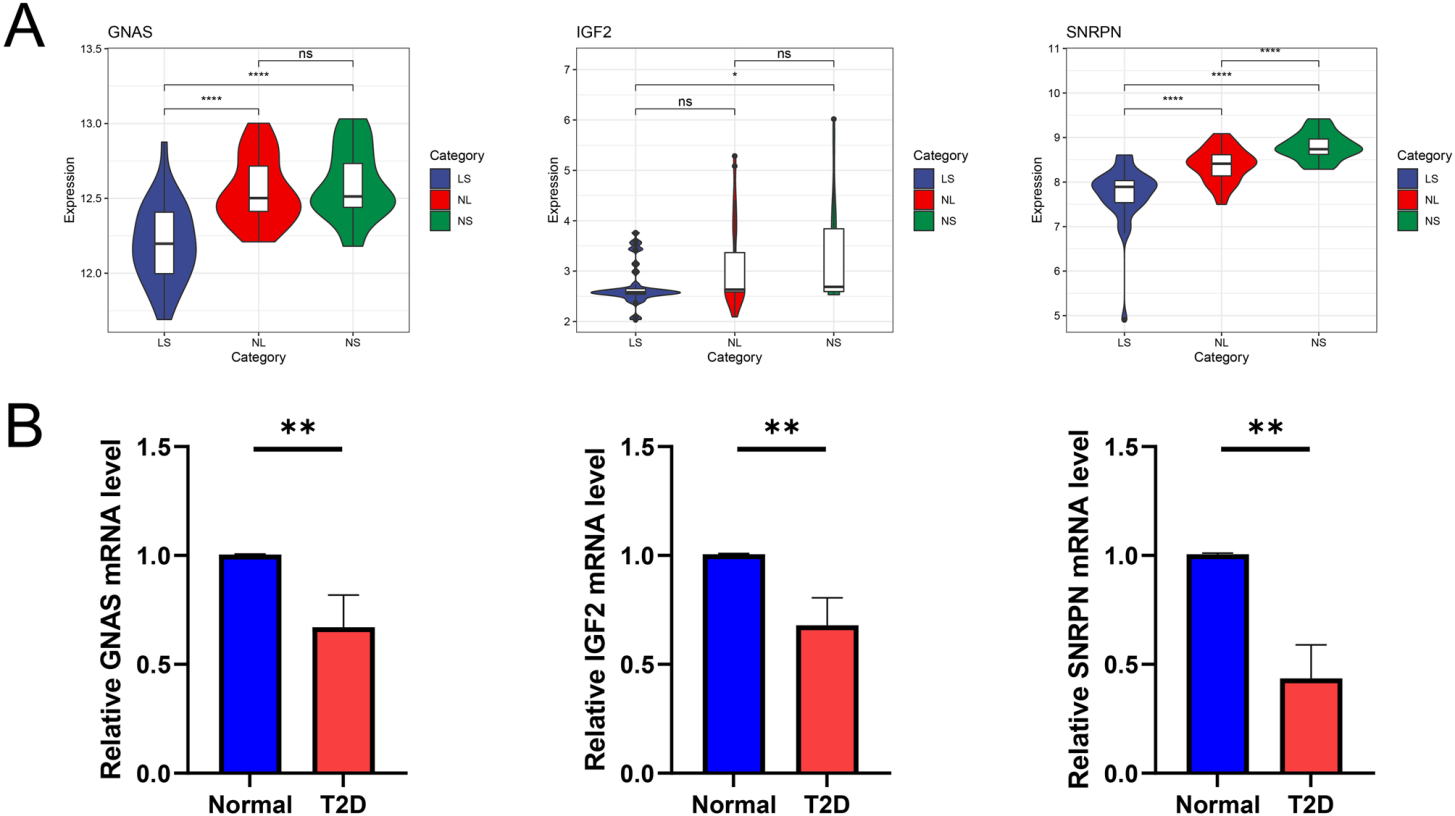
(**A**) The expression levels of the hub genes in GSE14905. A comparative analysis was conducted utilizing the mean *T*-test. A P-value of less than 0.05 was considered statistically significant. *LS* skin lesions, *NL* adjacent normal tissues, *NS*, normal skin. *p < 0.05; ***p < 0.001; ****p < 0.0001. (**B**) Three hub genes expression in patients with T2D and healthy control group by qRT-PCR.

To establish robustness of hub gene expression, we evaluated two additional datasets (GSE14905 and GSE38642) encompassing psoriasis lesions and type 2 diabetes and scrutinized the expression levels of the above hub genes. The comparison between healthy skin and psoriatic skin lesions revealed a significant downregulation in the expression of all hub genes (Fig. 7A). Furthermore, RT-qPCR experiments were performed to detect hub genes expression. As displayed in Fig. 7B, expression levels of SNRPN, GNAS and IGF2 in T2D patients were lower than those of healthy individuals. We also performed ROC curve analysis. And the result displayed that three hub genes (GNAS, AUC = 0.709; IGF2, AUC = 0.644; and SNRPN, AUC = 0.926) had a credible diagnostic value for psoriasis (Supplementary Fig. 1A). Subsequently, we verified the diagnostic value of these three hub genes in the GSE14905 dataset (GNAS, AUC = 0.692; IGF2, AUC = 0.573; and SNRPN, AUC = 0.588) (Supplementary Fig. 1B). As for T2D, three hub genes (GNAS, AUC = 0.952; IGF2, AUC = 0.714; and SNRPN, AUC = 0.905) also had a favorable diagnostic value (Supplementary Fig. 1C). The diagnostic value of these hub genes was validated using the GSE38462 dataset (GNAS, AUC = 0.733; SNRPN, AUC = 0.648) (Supplementary Fig. 1D).

### Identification and verification of TFs

**Figure 8:**
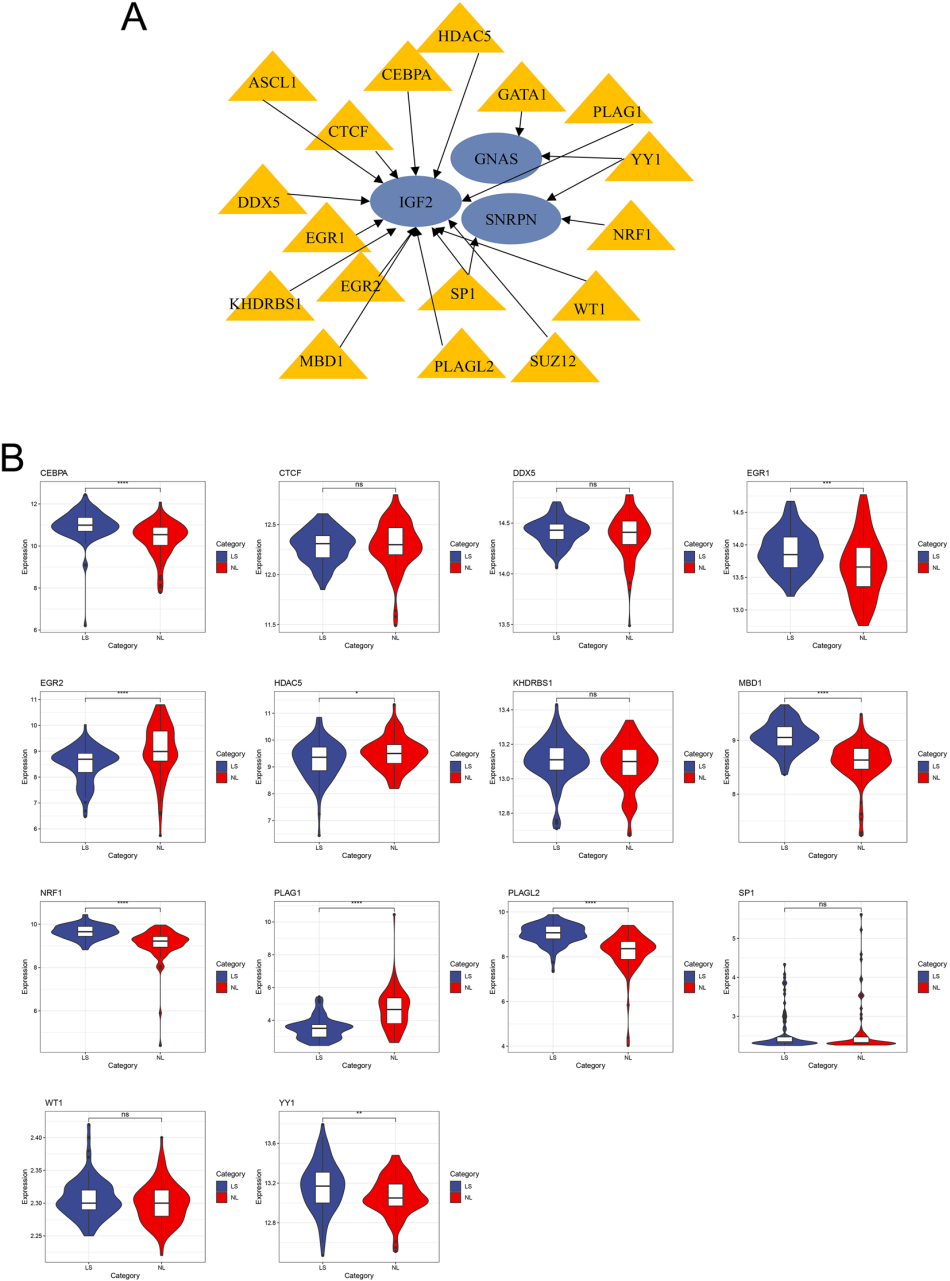
The regulatory network of TFs and their expression in GSE30999. (**A**) TFs regulatory network. TFs were marked in yellow, while the hub genes were marked in red. (**B**) Examination of TFs expression in GSE30999. A comparative analysis was conducted between the two datasets, utilizing the mean *T*-test. P-value of less than 0.05 was considered to represent a statistically significant difference. *LS* skin lesions, *NL* adjacent normal tissues, *AA* advanced atherosclerotic plaque, *EA* early atherosclerotic plaque samples. *p < 0.05; **p < 0.01; ****p < 0.0001.

**Figure 9:**
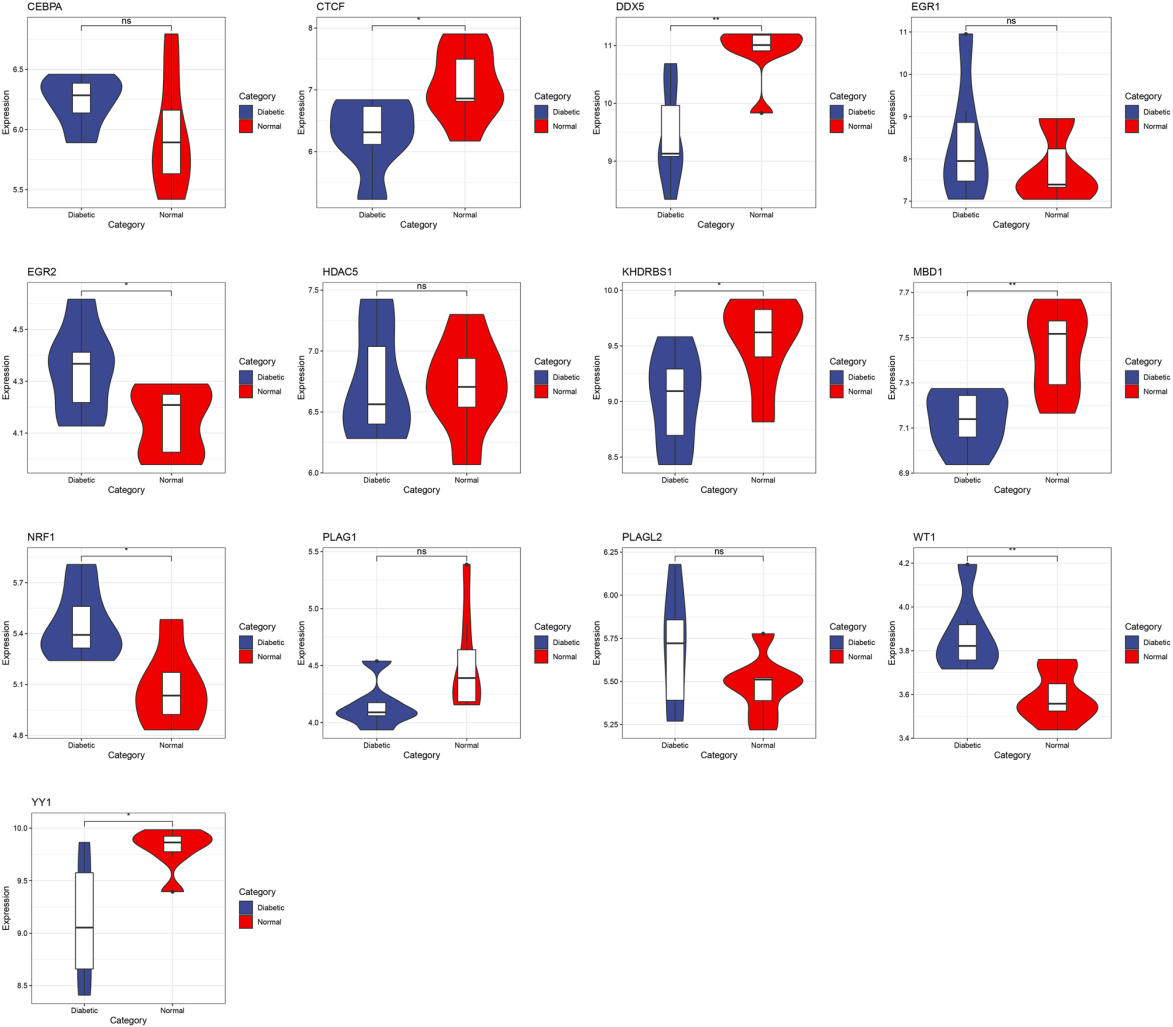
The level of TFs expression in the GSE25724 dataset. A comparative analysis was conducted between the two datasets, utilizing the mean *T*-test. A P-value of less than 0.05 was considered statistically significant. *p < 0.05; **p < 0.01.

Our analysis of the TRRUST database has allowed us to identify 17 transcription factors (TFs) that may potentially influence the regulation of gene expression specific to psoriatic lesions and T2D (Fig. 8A). Further validation of our findings revealed that amongst the identified TFs, only NRF1 exhibited significantly elevated expression level in psoriatic lesions (Fig. 8B) and T2D (Fig. 9). Notably, NRF1 predominantly modulated the expression of genes integral to antioxidant and detoxification pathways, with SNRPN being one of them.

## Discussion

Recent research has consistently highlighted a strong association between psoriasis and a high risk of major medical morbidities, and even mortality^20,21^. A cohort study found a connection between moderate and severe psoriasis and heightened chronic kidney disease risk^22^. An important positive dose–response correlation was found between psoriasis severity and uncontrolled hypertension^23^. The affected body surface area was conclusively determined to be a significant factor in both unadjusted and adjusted analyses, which took into account sex, age, alcohol use status, and other variables. Concurrently, a case–control study revealed that patients with psoriasis may experience more challenging-to-manage hypertension than non-psoriatic hypertensive patients^24^. A prospective, population-based cohort study provided evidence suggesting psoriasis as an independent risk factor for myocardial infarction^25^, with the highest relative risk observed in young patients suffering from severe psoriasis. Diabetes, currently the seventh leading cause of death in the United States, was initially linked as a comorbidity associated with psoriasis^26^. According to epidemiological studies, a significant association exists between T2D and psoriasis, with reported Odds Ratios (ORs) around 1.5^27,28^, even after accounting for body mass index (BMI) and other covariates. Patients with psoriasis have been found to exhibit diminished incretin effect^29^, and insulin response to oral glucose intake, suggesting a potential prediabetic condition. Importantly, patients exhibiting a higher degree of psoriasis severity were identified to have an increased risk of developing T2D^30^. A population-based study identified an adjusted odds ratio (OR) for diabetes of 1.13 (95% CI 1.08–1.18) for mild psoriasis and 1.62 (1.3–2.01) for severe psoriasis^4^.

The prevalent comorbidities seen in patients with chronic diseases, such as psoriasis and T2D, indicated a possible genetic link between these conditions^31–33^. Comprehensive genome-wide association studies have shed light on the genetic susceptibility loci specific to the psoriasis^34,35^ and T2D^36^ independently. However, studies investigating the mutual genetic markers of these diseases have been notably sparse^37^. Research conducted by Wang et al. studied the primary markers for 51 T2D loci in psoriasis cases versus controls, yielding two markers, located near ST6GAL1 and JAZF1 genes, with significant association with psoriasis among the Chinese population. Another investigation examined the genetic signals of T2D and psoriasis around the CDKAL1 locus in Caucasian patients. It concluded that, even though these signals are geographically proximate, their association is entirely independent (r^2^ = 0.04)^38^. Regardless, the molecular mechanism underlying the intricate interplay between psoriasis and T2D is yet to be completely understood. Our study first investigated the shared genes and related signaling pathways of psoriasis and T2D to guide earlier detection, better treatment, and timely prevention.

In our study, we pinpointed 132 DEGs common to psoriasis and T2D datasets. Our KEGG pathway enrichment analysis illustrated a correlation between these DEGs and salivary secretion. Past study has demonstrated reduced pilocarpine-stimulated salivary secretion in dermatitis-afflicted mice. Histological assessments of these mice revealed amyloid deposition, glandular atrophy, and fibrosis in their salivary glands^39^. Meanwhile, several epidemiologic studies have indicated that xerostomia is common among diabetes mellitus (DM) patients^40^. Furthermore, the DM population was found to present lower salivary flow rates than non-DM patients^41^. Renin-angiotensin system was overactivated owing to upregulated ACE expression in the psoriasis^42^. Similarly, early diabetes mellitus is characterized by renin-angiotensin system activation, and high levels of glucose levels directly activated the release of renin, a prohypertensive hormone^43^.

NF-κB signaling pathway was identified to be involved in the pathogenesis of psoriasis and T2D in past studies. In our research, three signaling pathways including PI3K-Akt, Rap1, and cGMP-PKG pathway were contained in enrichment results, suggesting that they seemed to be significant in the pathogenesis of psoriasis and T2D. The PI3K/Akt signaling pathway, key to aging and lifespan across organisms due to its capacity to significantly modify the activity and population of various stem cell types^44,45^, is strongly expressed in humans and mice psoriatic lesions. In the context of psoriasis, PI3K is observed to bind to Akt, leading to the subsequent activation of mTOR. This activation fosters keratinocyte hyperproliferation while inhibiting differentiation. Given its critical involvement in the etiology of psoriasis, the PI3K/Akt pathway is anticipated to be a potent target for anti-psoriatic targets. Recent findings have spotlighted topical rapamycin and delphinidin as promising therapeutic agents for psoriasis. These agents have been shown to mitigate psoriatic lesions in the imiquimod (IMQ)-induced psoriasis mouse model by suppressing the PI3K/Akt/mTOR pathway^46–48^. An increasing number of evidence has demonstrated that PI3K/Akt pathway played a crucial role in T2D, and it could be a promising therapeutic target for the treatment of T2D^49–51^. Rap1, a small monomeric GTPase, forms part of the broader Ras family^52^. Its existence in the body is a product of the encoding by two specific genes: Rap1a and Rap1b. The prevalence of Rap1 is ubiquitous, with expression observed throughout various regions of the body. Here, we have discovered an intriguing correlation: when Rap1 activity is selectively augmented within the medial hypothalamus, it causes an elevation of blood glucose levels. This observed increase is notable, especially as it occurs without any corresponding rise in body weight among mice that have been fed a high-fat diet^53^. We speculate that Rap1 pathway and the cGMP-PKG signaling pathway could represent novel targets for psoriasis and T2D treatment.

We first identified three hub genes, including SNRPN, IGF2, and GNAS, which could be potential therapeutic targets of psoriasis and T2D. Our ROC analysis result also demonstrated that these three hub genes had credible diagnosis values in psoriasis and T2D. Located within the 15q11-q13 region of chromosome 15, the SNRPN gene encodes the RNA-binding SmN protein. This specific region is implicated in a number of neurodevelopmental disorders, including Prader-Willi syndrome (PWS), angelman syndrome (AS), and autism spectrum disorders (ASDs)^54^. Interestingly, The expression of the SNRPN gene varies across different tissues, but it predominantly displays the highest levels in the adult brain and heart^55^. The IGF2 gene, or Insulin-like growth factor 2, synthesized and released by adult β-cells, acts as an autocrine stimulator for insulin-like growth factor 1 receptor signaling pathway of the β-cells. This auto-regulatory function of IGF2 is paramount in regulating adult β-cell mass and functionality, thereby preserving the physiological mechanism of glucose-stimulated insulin secretion^56^. Accumulating evidence has revealed that IGF-2 messenger RNA (mRNA)-binding protein 2 (IGF2BP2) was involved in the pathogenesis of T2DM by regulating glucose metabolism and insulin sensitivity^57^. The GNAS gene encodes the Gαs protein, which plays a crucial role in facilitating G protein-coupled receptor (GPCR) signaling. Mutations in GNAS can result in developmental delay, short stature, and skeletal abnormalities associated with Albright’s hereditary osteodystrophy. Mutations on the maternally inherited allele can also lead to obesity and hormone resistance, characteristic of pseudohypoparathyroidism, due to the effects of genetic imprinting^58^.

In conclusion, our study pioneered the identification and examination of shared DEGs, central hub genes, and TFs between psoriasis and T2D. This novel exploration significantly contributed to our comprehension of the molecular mechanisms that underpin these conditions. However, we acknowledged certain limitations within our study. Primarily, the retrospective nature of our research required further validation through external corroboration to confirm our findings. Additionally, the identified hub gene’s functional relevance required more comprehensive analysis, ideally within an in vitro setting. These aspects presented themselves as the focus of our future investigation.

### Supplementary Information

Supplementary Figure S1.

## Data Availability

The data which support the findings of our work are openly accessible from the GEO, STRING, GeneMANIA, and Cytoscape database.
