# Supplementary figures and images for: Exploring the molecular mechanism underlying the psoriasis and T2D by using microarray data analysis
Source: Sci Rep. 2023 Nov 7;13:19313. doi: 10.1038/s41598-023-46795-5 (PMC10630520; doi:10.1038/s41598-023-46795-5)

**Supplementary Material**

**FIGURE S1**


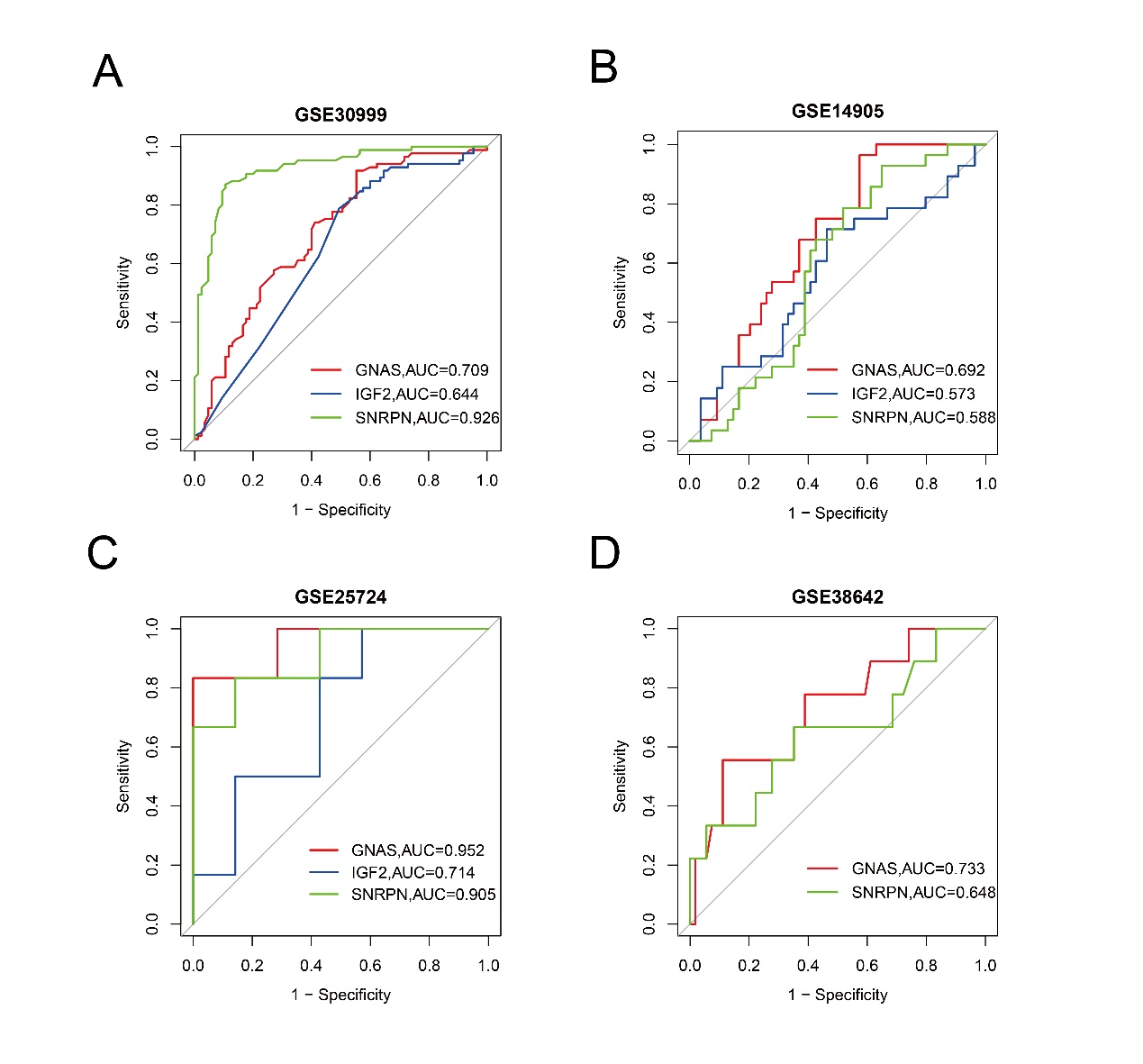


**FIGURE S1**

The ROC curve of three genes in the (A) GSE30999, (B) GSE14905, (C) GSE25724, (C) GSE38642.

Supplement: Supplementary file 1 — Supplementary Figure S1. [file 41598_2023_46795_MOESM1_ESM.docx]
